# Promising 8-Aminoquinoline-Based Metal Complexes in the Modulation of SIRT1/3-FOXO3a Axis against Oxidative Damage-Induced Preclinical Neurons

**DOI:** 10.1021/acsomega.3c06764

**Published:** 2023-11-30

**Authors:** Waralee Ruankham, Napat Songtawee, Veda Prachayasittikul, Apilak Worachartcheewan, Wilasinee Suwanjang, Ratchanok Pingaew, Virapong Prachayasittikul, Supaluk Prachayasittikul, Kamonrat Phopin

**Affiliations:** † Center for Research Innovation and Biomedical Informatics, Faculty of Medical Technology, Mahidol University, Bangkok 10700, Thailand; ‡ Department of Clinical Chemistry, Faculty of Medical Technology, Mahidol University, Bangkok 10700, Thailand; § Department of Community Medical Technology, Faculty of Medical Technology, Mahidol University, Bangkok 10700, Thailand; ∥ Department of Chemistry, Faculty of Science, Srinakharinwirot University, Bangkok 10110, Thailand; ⊥ Department of Clinical Microbiology and Applied Technology, Faculty of Medical Technology, Mahidol University, Bangkok 10700, Thailand

## Abstract

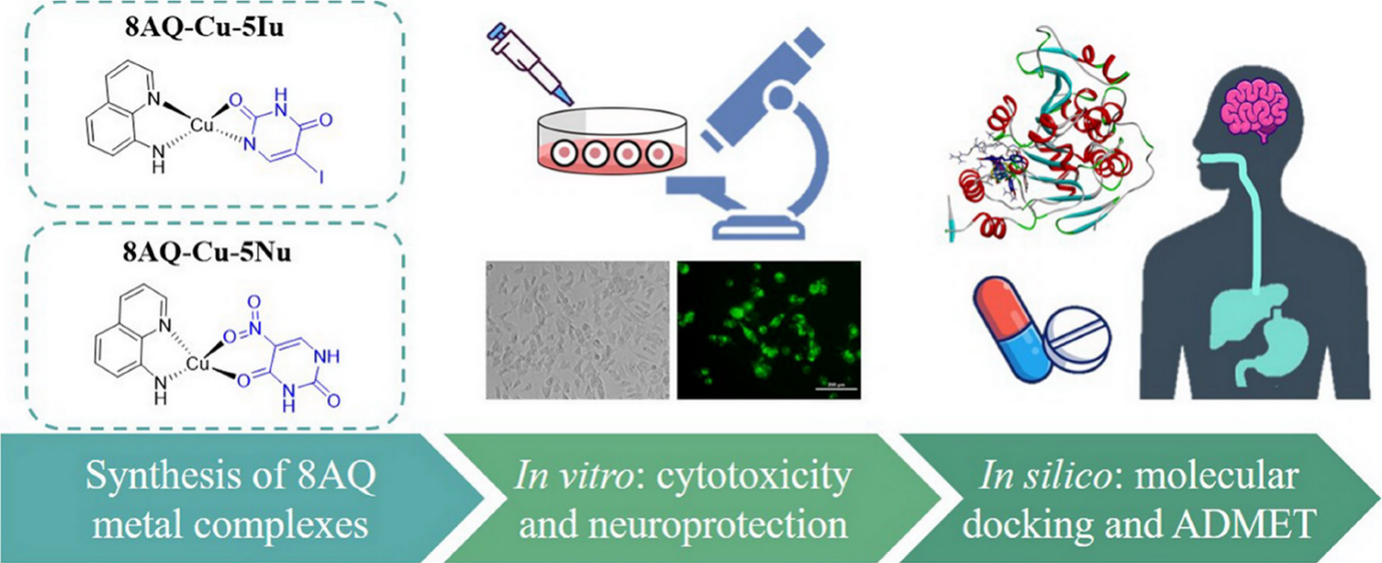

The discovery of novel bioactive molecules as potential multifunctional neuroprotective agents has clinically drawn continual interest due to devastating oxidative damage in the pathogenesis and progression of neurodegenerative diseases. Synthetic 8-aminoquinoline antimalarial drug is an attractive pharmacophore in drug development and chemical modification owing to its wide range of biological activities, yet the underlying molecular mechanisms are not fully elucidated in preclinical models for oxidative damage. Herein, the neuroprotective effects of two 8-aminoquinoline–uracil copper complexes were investigated on the hydrogen peroxide-induced human neuroblastoma SH-SY5Y cells. Both metal complexes markedly restored cell survival, alleviated apoptotic cascades, maintained antioxidant defense, and prevented mitochondrial function by upregulating the sirtuin 1 (SIRT1)/3-FOXO3a signaling pathway. Intriguingly, *in silico* molecular docking and pharmacokinetic prediction suggested that these synthetic compounds acted as SIRT1 activators with potential drug-like properties, wherein the uracil ligands (5-iodoracil and 5-nitrouracil) were essential for effective binding interactions with the target protein SIRT1. Taken together, the synthetic 8-aminoquinoline-based metal complexes are promising brain-targeting drugs for attenuating neurodegenerative diseases.

## 1 Introduction

Neurodegeneration is a chronic progressive condition that causes gradual cognitive impairment and physical disability, resulting in consequentially devastating dementia. With an unprecedented increase in the population of older individuals worldwide, ∼130 million older individuals are expected to be living with dementia in 2050.^1,2^ Furthermore, the recent global coronavirus disease 2019 (COVID-19) pandemic reportedly prompted numerous neurological sequelae and increased mortality associated with neurodegenerative diseases.^3^ The earliest deterioration of excessive reactive oxygen species (ROS) accumulation alongside impaired antioxidant defenses has been considered to destroy cellular components and genetic materials in pathophysiological neurodegeneration, leading to neuronal dysfunction, oxidative damage, and ultimately apoptotic cascades.^4−6^ Besides the pivotal role of oxidative stress, transition metal ions play essential roles in biological processes owing to their redox nature, which forms a key feature of their reactivity. The loss of metal ion homeostasis in the brain could be one of the leading factors inducing oxidative stress conditions, i.e., redox homeostasis loss, oxidative damage, and amyloid-β (Aβ) plaque deposition and aggregation in Alzheimer’s disease (AD). Among all the ions, the decline of intracellular copper (Cu) levels critically affects neuronal functions.^7^

Quinoline derivatives are well recognized for their metal-chelating properties; thus, they are widely used as pharmacophores in medicinal chemistry.^8^ The most promising scaffold, 8-hydroxyquinoline (8HQ), has been represented by its multiple therapeutic effects.^9^ Particularly, clioquinol (PBT1) and PBT2-based 8HQ exhibit neuroprotective effects in cognitive transgenic mice models as well as in clinical trials by acting as Cu/zinc ionophores.^8,10,11^ Although these compounds facilitate metal uptake into the brain, the metal affinity remains limited when compared with that of metalloproteins.^12,13^ This has led to the search for Cu-complexing compounds, which exhibit greater effectiveness in targeting and restoring metal homeostasis in the brain of patients with AD. Consequently, the PBT2-based Cu complex was designed to provide preferable and better selective chelating properties for regulating Cu homeostasis.^14^ The intensive search for drug discovery has been extended to the related quinoline class, aminoquinoline. Aminoquinoline-based analogues are the only class of drugs approved by the Food and Drug Administration for the hepatic treatment of patients with *Plasmodium vivax* and *Plasmodium ovale* infections.^15^ As previously reported, a set of mixed ligands 8-aminoquinoline (8AQ)–uracil metal complexes demonstrated promising biological properties, including anticancer against human leukemia T-lymphocyte (MOLT-3) cells,^16^ preferable superoxide scavenging activity,^16^ cytotoxicity against normal embryonic lung (MRC-5) cells,^17^ and aromatase inhibitory.^17^ These 8AQ-based metal complexes also demonstrated more potent antimalarial and antimicrobial activities than those of their parent 8AQ.^15^ Regarding their antioxidant properties, this set of 8AQ-based metal complexes is notable and should be explored for their neuroprotective potentials.

Herein, two previously reported 8AQ-based Cu complexes (8AQ–Cu–5Iu and 8AQ–Cu–5Nu) were investigated for their neuroprotective effects against oxidative stress-induced human SH-SY5Y neuroblastoma cells. Cell viability was assessed using a 3-(4,5-dimethylthiazol-2-yl)-2,5-diphenyltetrazolium bromide (MTT) assay. Subsequently, apoptotic profiles, intracellular ROS levels, and mitochondrial membrane potential (MMP) were determined. The possible underlying mechanism of this neuroprotective effect was explored by using Western blotting. Furthermore, molecular docking was conducted to reveal the binding modes and key features between the synthetic compounds and the longevity sirtuin-1 (SIRT1) protein. *In silico* pharmacokinetic prediction, i.e., absorption, distribution, metabolism, excretion, and toxicity (ADMET), was performed to ensure drug-likeness and the pharmacological possibility to be further developed.

## 2 Results

### 2.1 8AQ-Based Metal Complexes Improve Cell Viability in H_2_O_2_-Induced SH-SY5Y Cells

**Figure 1:**
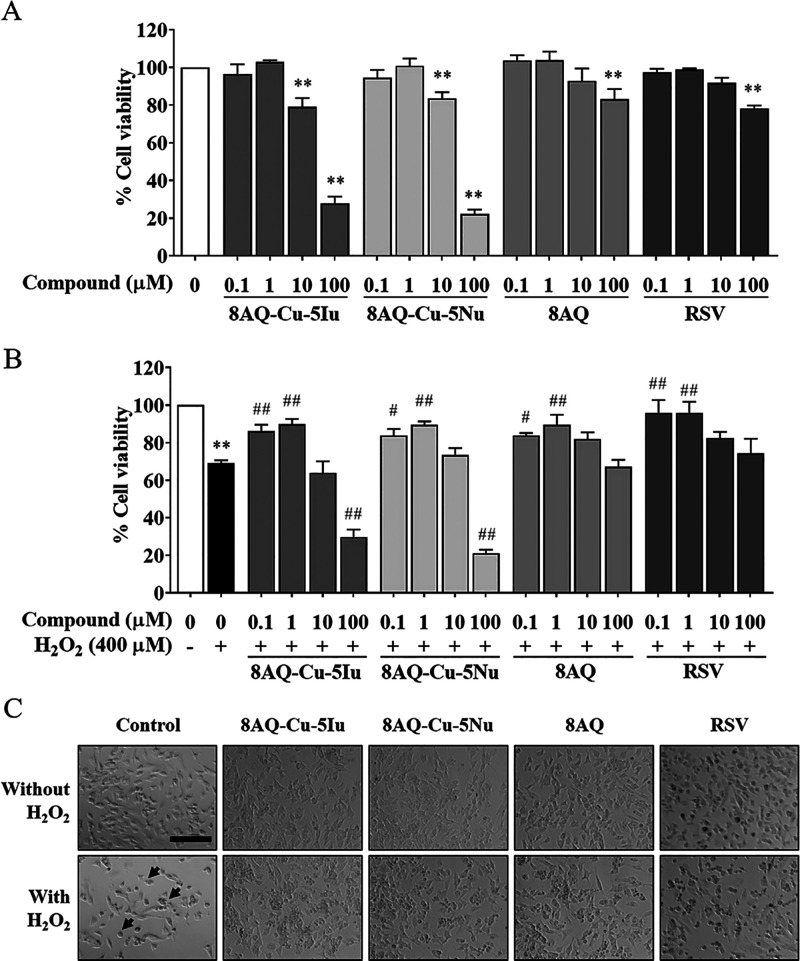
8AQ-based complexes did not induce cytotoxicity at concentrations of 0.1 and 1 μM. (A) Effect of 8AQ–Cu–5Iu, 8AQ–Cu–5Nu, and 8AQ on the viability of SH-SY5Y cells was assessed using the MTT assay. (B) 8AQ-based complexes significantly increased the viability of H_2_O_2_-treated SH-SY5Y cells. (C) Morphological changes induced by pretreating the oxidative stress-induced SH-SY5Y cells with the test compounds were captured at a magnification of ×20 (bar = 500 μm in length). Arrows indicate the morphological changes associated with apoptosis. Data are represented as means ± SEM of three independent experiments. **P < 0.01 compared with the untreated cells; ^#^*P* < 0.05 and ^##^*P* < 0.01 compared with the H_2_O_2_ group.

To potentiate the promising neuroprotective effect of 8AQ metal complexes, experimental and biological studies were conducted. A well-known antioxidant agent, resveratrol (RSV), was used as a positive control. Cytotoxicity was screened via a reduction of colorimetric assay by observing intracellular metabolic activity. The results of the MTT assay revealed that the tested metal complexes, parent ligand 8AQ, and reference compound RSV did not alter cell viability at concentrations of 0.1 and 1 μM. At a higher concentration of 10 μM, the metal complexes induced substantial cell death, whereas 8AQ and RSV exhibited no cytotoxicity (Figure 1A). For the neurodegenerative disease model, 400 μM hydrogen peroxide (H_2_O_2_) was used to mimic oxidative stress in human SH-SY5Y cells. H_2_O_2_ treatment induced significant cell death, which was 31% of that of untreated cells. Pretreating with 0.1 and 1 μM tested compounds (8AQ–Cu–5Iu, 8AQ–Cu–5Nu, and 8AQ ligand) considerably prevented the decrease of cell viability in H_2_O_2_-treated SH-SY5Y cells and increased cell viability by ∼80% compared with the RSV (Figure 1B). H_2_O_2_ treatment induced morphological changes, including cellular shrinkage, shortened neurites, and smaller vesicular bodies compared with the control. Conversely, pretreating with the tested compounds (8AQ–Cu–5Iu, 8AQ–Cu–5Nu, and 8AQ ligand) protected the cells from changing into apoptotic neurons in comparison to those in the H_2_O_2_-treated group (Figure 1C).

### 2.2 Pretreatment of 8AQ-Based Metal Complexes Attenuates Apoptotic Profiles against H_2_O_2_-Treated SH-SY5Y Cells

**Figure 2:**
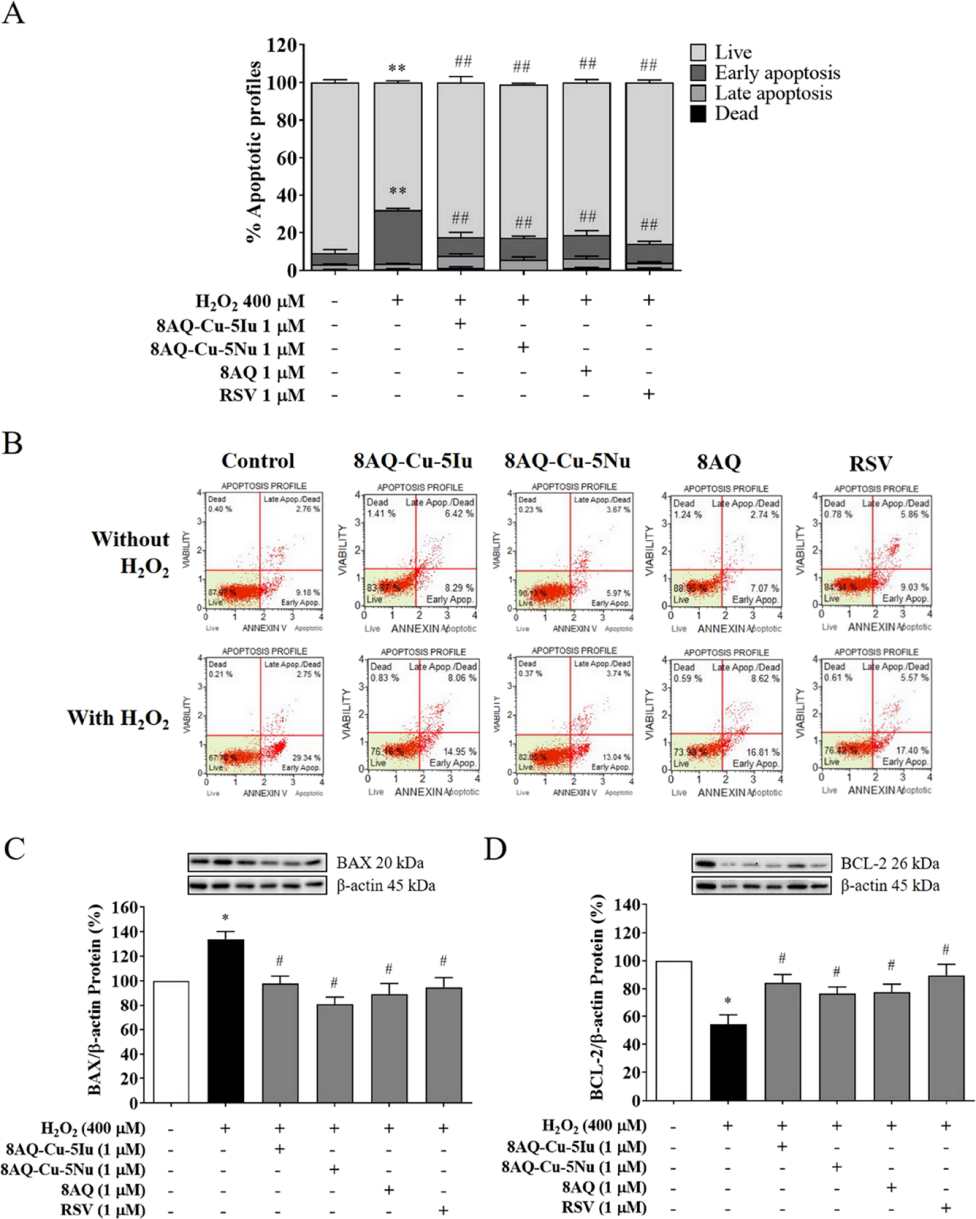
8AQ-based metal complexes significantly reduced the total percentage of apoptosis. (A) Percentage of the apoptotic profiles in SH-SY5Y cells was quantified using flow cytometry. (B) Scattergram of 8AQ–Cu–5Iu, 8AQ–Cu–5Nu, and the 8AQ ligand is represented in various manners: viable (lower left), early apoptosis (lower right), late apoptosis (upper right), and dead cells (upper left). (C) BAX and (D) BCL-2 protein expression levels of the 8AQ metal complexes induced by H_2_O_2_ exposure were evaluated using Western blotting. Data are represented as means ± SEM of three independent experiments. **P < 0.01 compared with the untreated cells; ^##^*P* < 0.01 compared with the H_2_O_2_ group.

The protective effect of the test compounds (8AQ–Cu–5Iu, 8AQ–Cu–5Nu, and 8AQ ligand) against H_2_O_2_-induced apoptosis in SH-SY5Y cells was investigated. The 8AQ metal complex-pretreated SH-SY5Y cells were treated with 400 μM H_2_O_2_ before flow cytometry and Western blotting analyses. The results indicated that exposure to 400 μM H_2_O_2_ significantly increased the percentage of apoptotic cells by ∼40% in the SH-SY5Y cells compared with that in the untreated cells. Pretreatment with 1 μM 8AQ–Cu–5Iu, 8AQ–Cu–5Nu, and 8AQ considerably reduced the total percentage of apoptosis in the cells from early to late apoptosis phases by 19–21% (Figure 2A) as shown in the scattergram of apoptotic profile reduction (Figure 2B). To explore the insight of molecular protein-targeted apoptotic cascades, Western blotting was employed to determine the protein expression against the oxidative stress model. H_2_O_2_ exposure substantially increased the expression levels of the proapoptotic protein BAX up to 34% (Figure 2C), while decreasing those of the antiapoptotic B-cell lymphoma 2 (BCL-2) protein by 46% compared with the control group (Figure 2D). Conversely, pretreatment with RSV restored the homeostasis of these proteins (Figure 2C,D). Additionally, pretreatment with the tested compounds and RSV recovered the imbalance of the apoptotic pathways in H_2_O_2_-treated cells (Figure 2C,D). These results suggest that the tested 8AQ-based complexes and parental ligand can improve neuronal survival in H_2_O_2_-induced apoptosis in SH-SY5Y cells.

### 2.3 Intracellular ROS Generation and MMP Changes Are Maintained by 8AQ-Based Metal Complexes-Pretreated SH-SY5Y Cells

**Figure 3:**
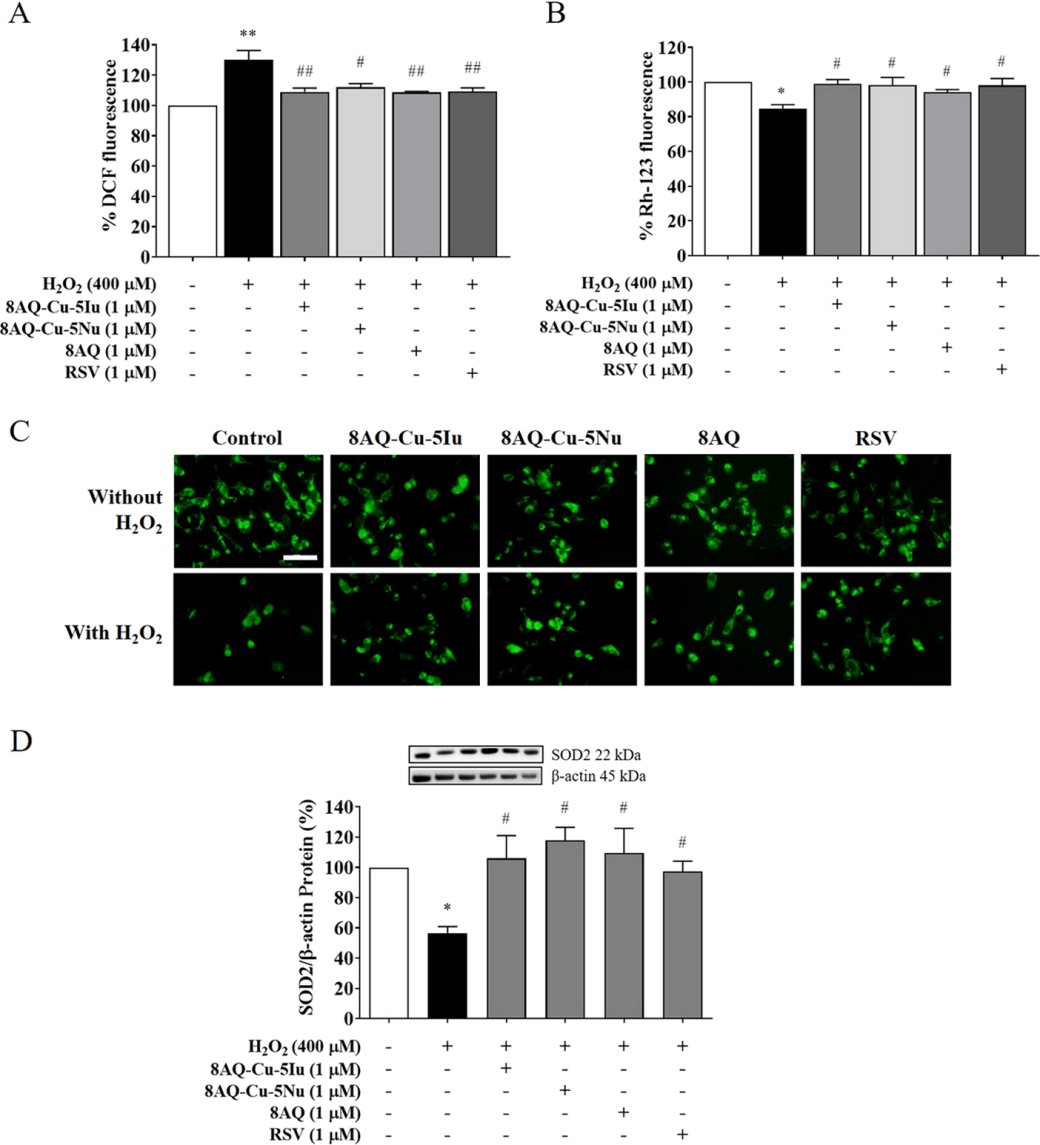
Antioxidant defense systems and mitochondrial statuses were maintained by 8AQ-based metal complexes. The effects of 8AQ–Cu–5Iu, 8AQ–Cu–5Nu, and 8AQ pretreatments against H_2_O_2_-treated SH-SY5Y cells were determined via ROS production and MMP level using (A) dichlorodihydrofluorescein diacetate assay and (B) rhodamine staining. (C) Fluorescent MMP signals of the 8AQ metal complexes-pretreated cells were imaged. (D) Protein expression levels of SOD2 were measured using Western blotting. Data are represented as means ± SEM of three independent experiments. *P < 0.05 and **P < 0.01 compared with the untreated cells; ^#^*P* < 0.05 and ^##^*P* < 0.01 compared with the H_2_O_2_ group.

The effects of 8AQ metal complexes and their parent ligand (8AQ) on intracellular ROS production and mitochondrial function against H_2_O_2_-induced oxidative stress in neurons were investigated. H_2_O_2_ exposure significantly increased ROS production (30%) (Figure 3A), and the loss of MMP was below that of the control (15%) (Figure 3B). Particularly, the change in MMP was confirmed via green fluorescent rhodamine monitoring (Figure 3C). As expected, pretreatments with 8AQ–Cu–5Iu, 8AQ–Cu–5Nu, and 8AQ decreased ROS production and increased MMP levels, thereby indicating its abilities to improve those neuropathological conditions to the same extent as the antioxidant positive control, RSV (Figure 3A–C). Moreover, exogenous H_2_O_2_ exposure also disrupted manganese superoxide dismutase (SOD2) protein expression levels by ∼43% compared with those of the untreated cells. However, pretreatment with 8AQ–Cu–5Iu, 8AQ–Cu–5Nu, and 8AQ considerably upregulated SOD2 protein expression levels in comparison to those in the H_2_O_2_-treated group (Figure 3D). This could be due to the potent antioxidant potential of these metal–8AQ complexes, which is slightly higher than that of the RSV. Therefore, the antioxidant property of the tested metal–8AQ complexes may endow them with abilities to reduce intracellular ROS levels, maintain mitochondrial function, and restore the neuronal oxidant–antioxidant system.

### 2.4 8AQ-Based Metal Complexes Mediate Cell Survival through the SIRT1/3-FOXO3a Signaling Pathway

**Figure 4:**
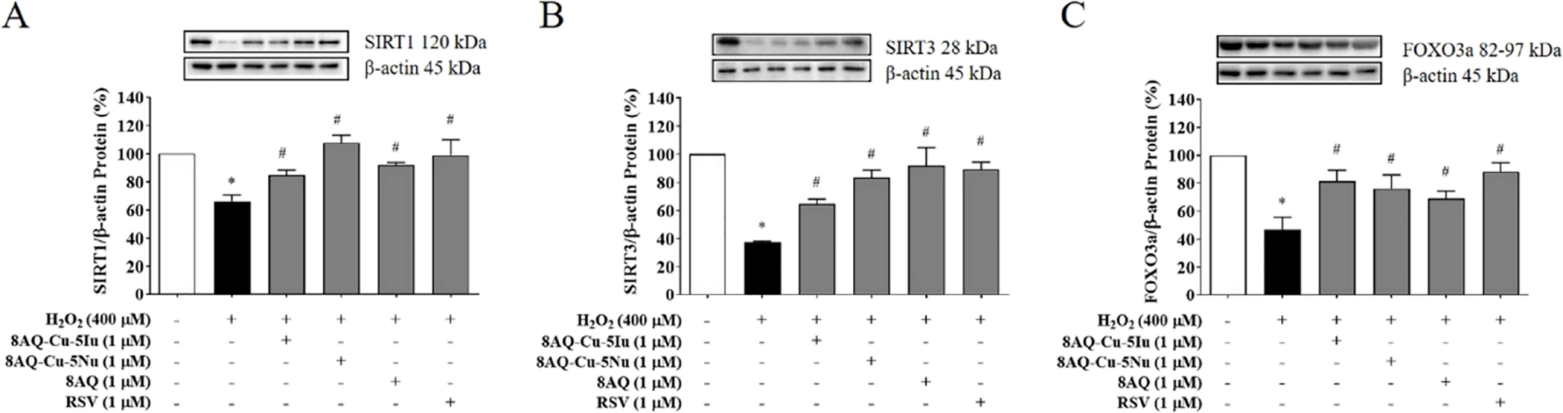
Neuroprotective effects of 8AQ-based metal complexes by regulating the SIRT1/3-FOXO3a signaling pathway were investigated in the H_2_O_2_-treated SH-SH5Y cells pretreated with the tested compounds. The protein expression levels were evaluated via Western blotting, including those of (A) SIRT1, (B) SIRT3, and (C) FOXO3a. Data are represented as means ± SEM of three independent experiments. *P < 0.05 compared with the untreated cells; ^#^*P* < 0.05 compared with the H_2_O_2_ group.

To further evaluate whether the identifying compounds can modulate the neuroprotective effects against H_2_O_2_-induced oxidative damage, the present study focused on the SIRT1/3 signaling pathway. As shown in Figure 4A,B, the H_2_O_2_-exposed cells demonstrated substantially decreased SIRT1 (34%) and SIRT3 (63%) expression levels compared with those of the unexposed control cells. Conversely, pretreatment with the tested compounds (8AQ–Cu–5Iu, 8AQ–Cu–5Nu, and 8AQ) attenuated SIRT1 and SIRT3 expression levels comparable with that attenuated by the known SIRT1/3 activator RSV. Similarly, a decrease in the expression levels of forkhead box class O3a (FOXO3a) by 53% was observed in the H_2_O_2_-treated cells compared with that of the untreated group, whereas the expression levels were restored in the cells pretreated with the tested compounds (8AQ–Cu–5Iu, 8AQ–Cu–5Nu, and 8AQ), similar to the trend exhibited by RSV (Figure 4C). It could be suggested that the tested 8AQ metal complexes can positively modulate the SIRT1/3-FOXO3a signaling pathway.

### 2.5 8AQ-Based Metal Complexes Act as SIRT1 Activators

**Figure 5:**
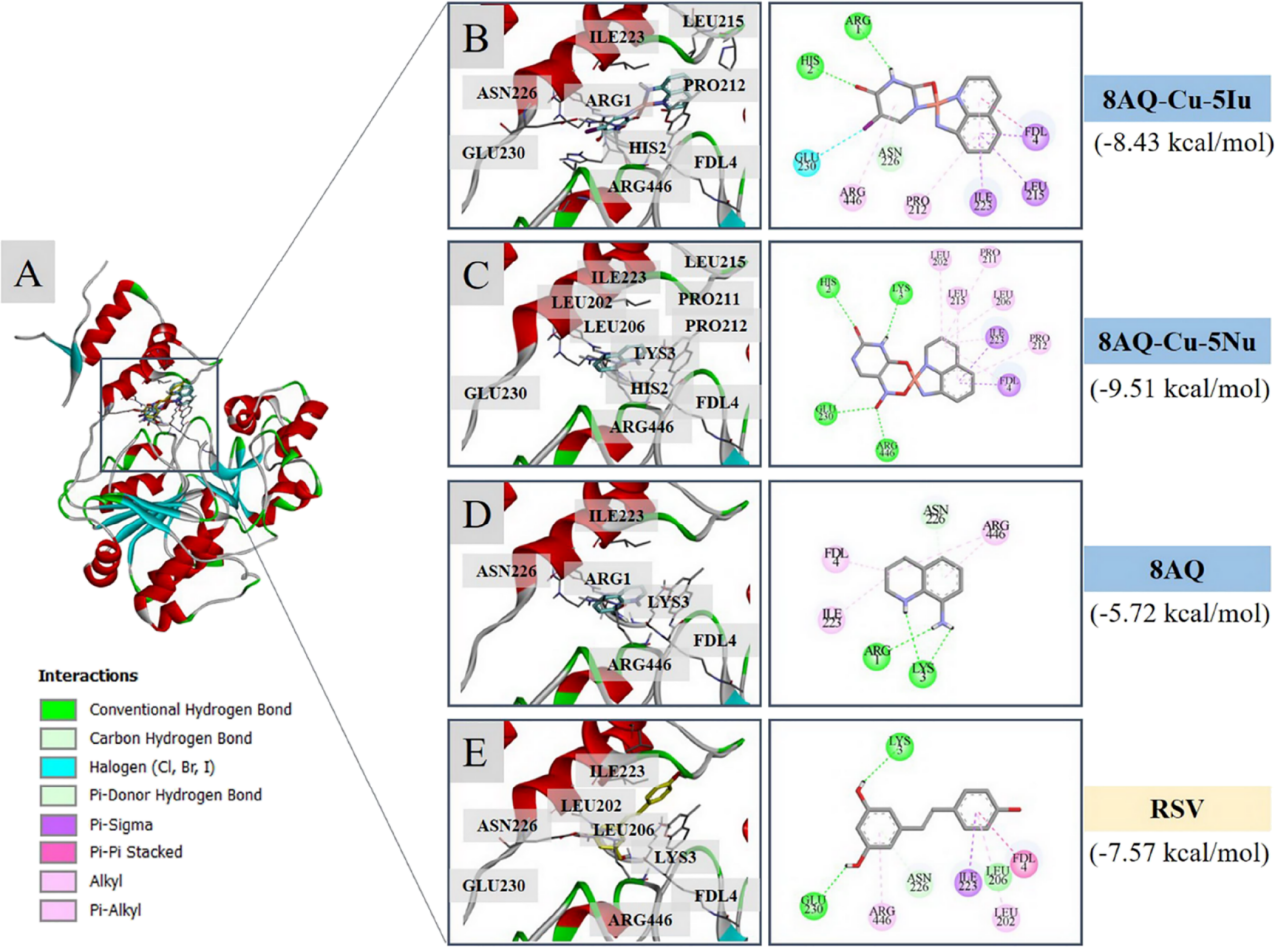
Binding modalities of the investigated 8AQ-based metal complexes against the target SIRT1 (PDB: 5BTR). (A) Redocking poses; redocked RSV pose (yellow) concerning the cocrystallized RSV (gray), and docking poses of the tested compounds (8AQ–Cu–5Iu and 8AQ–Cu–5Nu) within the SIRT1 activator binding site. Two-/three-dimensional ligand–protein interaction diagrams of (B) 8AQ–Cu–5Iu, (C) 8AQ–Cu–5Nu, (D) parent ligand 8AQ, and (E) RSV.

Molecular docking study against the target protein SIRT1 (retrieved in complex with RSV and an AMC-containing peptide, PDB ID: 5BTR) was performed to reveal the possible binding modalities of the metal complexes. Initially, redocking was performed using a cocrystallized ligand (RSV) to validate the reproducibility of the docking protocol, and root-mean-square deviation (RMSD) was calculated; an RMSD value of <2.0 Å indicated the reliability of the simulation for further investigation.^18^ The calculated RMSD value was 0.57 Å, which confirmed that the protocol was acceptable for further compound investigation. The studied metal complexes were subsequently docked into the active site of SIRT1 to reveal their binding modes. The results demonstrated that all of the metal complexes and parent ligand 8AQ occupied the binding site of SIRT1 in the same manner as that of the SIRT1 activator RSV (Figure 5A). The binding free energies of the metal complexes 8AQ–Cu–5Iu (−8.43 kcal/mol) and 8AQ–Cu–5Nu (−9.51 kcal/mol) were relatively lower than those of the original ligand (8AQ, −5.72 kcal/mol) and the reference (RSV, −7.57 kcal/mol). The ligand–protein interaction diagrams revealed that both metal complexes bind to the target via the formation of hydrogen bonds (green), π-type interactions (magenta), and halogen interactions (blue) (Figure 5B–E). Two metal complexes and the 8AQ ligand shared some common key interacting amino acid residues with RSV, including the residues within both N-(GLU230, ILE223, and ASN226) and C-(ARG446) terminals of SIRT1 as well as those located on the fluorogenic peptide (LYS3). Interaction with GLU230 is absent in the binding of the parent 8AQ ligand compared with that in the RSV (Figure 5D,E).

### 2.6 8AQ-Based Metal Complexes Provide Potential Drug-like Properties for Restoring Metal Imbalance

To assess the physicochemical and pharmacokinetic (drug-like) properties of the 8AQ-based metal complexes, the ADME parameters were computed using the SwissADME open-access Web site.^19^ In addition, pkCSM, an online tool, was used to calculate the parameters regarding cytotoxicity.^20^ The predicted ADMET profiles of the tested metal complexes (8AQ–Cu–5Iu, 8AQ–Cu–5Nu), as well as the parental ligand (8AQ), are shown in Table 1. Based on Lipinski’s rule of five, the prediction values suggested that the tested compounds fall within all requirements without violating the Lipinski’s rule.^21,22^ To strongly support the *in silico* predictions of Lipinski’s rule, Veber’s rules^23^ were further applied and the theoretical calculation found that these compounds displayed preferable bioavailability and intestinal absorption, as indicated by their molar refractivity values (between 40 and 130) alongside their molecular flexibility (rotatable bonds <10). Particularly, 8AQ–Cu–5Iu, 8AQ–Cu–5Nu, and 8AQ were predicted to display high gastrointestinal absorption potential (as indicated by a polar surface area value of <140 Å^2^)^24^ as well as preferable bioavailability; however, they were predicted to exhibit poor skin penetrating properties (as revealed by low permeability coefficient [Kp] values).^25^ Interestingly, these complexes could pass across the blood–brain barrier (BBB) in addition to the calculated Log BB values. The tested compounds were also predicted to act as CYP1A2 inhibitors, which suggests their potentials to produce drug–drug/food–drug interactions. Lastly these compounds exhibited better selectivity toward the CYP450 family compared with that of the RSV (an inhibitor against three isoforms of CYP450, i.e., CYP1A2, CYP2C9, and CYP3A4).

**Table 1:** Predicted Physicochemical and Pharmacokinetic Properties of 8AQ-Based Metal Complexes, 8AQ Ligand, and RSV

property	8AQ–Cu–5Iu	8AQ–Cu–5Nu	8AQ	RSV
physicochemical properties				
formula	C_13_H_9_CuIN_4_O_2_	C_13_H_10_CuN_5_O_4_	C_9_H_8_N_2_	C_14_H_12_O_3_
molecular weight	443.69	363.8	144.17	228.24
rotatable bonds	0	0	0	2
H-bond acceptors	2	4	1	3
H-bond donors	2	3	1	3
polar surface area	71.82	128.5	38.91	60.69
lipophilicity	2.77	–0.23	1.79	3.13
molar refractivity	87.56	87.95	46.15	67.88
pharmacokinetic properties				
water solubility	soluble	soluble	soluble	slightly soluble
GI absorption	high	high	high	high
skin permeability	low	low	low	low
BBB permeability	yes	no	yes	yes
bioavailability	0.55	0.55	0.55	0.55
CYP1A2 inhibitor	yes	yes	yes	yes
CYP2C19 inhibitor	no	no	no	no
CYP2C9 inhibitor	no	no	no	yes
CYP2D6 inhibitor	no	no	no	no
CYP3A4 inhibitor	no	no	no	yes

## 3 Discussion

Although there are several clinical drugs available for neurodegenerative treatment. Most of these drugs are symptomatic, as well as their therapeutic efficacy, pharmacokinetics, and toxic profiles remain to be addressed. Accordingly, the development of novel neuroprotective agents is an ongoing research area. Recently, metal-based compounds and complexes have gained considerable attention in the field of drug discovery owing to their multifunctional nature and preferable pharmacokinetic profiles. Numerous types of metal-based compounds have been developed for the treatment of cancer,^26^ severe acute respiratory syndrome coronavirus 2 infection,^27^ and neurodegeneration.^28^ The free radical scavenging characteristic of metal complexes renders them one of the promising classes of candidates for protection against neurodegeneration.^29,30^ Evidence from preclinical studies showed that our 8AQ–uracil Cu complexes (8AQ–Cu–5Iu and 8AQ–Cu–5Nu) and RSV exhibited no neurotoxicity (at concentrations <100 μM), which could be employed to investigate signaling cascades within human neuroblastoma SH-SY5Y cells. These biphasic behaviors of RSV have been demonstrated on both tumor cell lines and normal cells.^31,32^ It is increasingly interesting that the RSV can scavenge ROS at low concentrations, while it exhibits apoptosis like a pro-oxidant at higher concentrations. With other previous supports, the results corroborated that multiple effects of RSV were decided by its concentration-dependent manner. The dual effects of RSV could be referred to our 8AQ–uracil Cu complexes, for which further studies are required. H_2_O_2_ was selected to mimic the oxidative stress conditions and mitochondrial dysfunction in SH-SY5Y cells. The related quinoline derivative, 6-hydroxy-2,2,4-trimethyl-1,2-dihydroquinoline (DHQ), reportedly increases cell survival in cerebral ischemia rat models by reducing the levels caspase-3/8 and apoptosis-inducing factor.^33^

The mitochondrial content of the central nervous system (CNS) serves as the main source of adenosine triphosphate (ATP) and the proper oxygen supply that drives numerous biological processes in the neurons. Considering the limited regenerative capacity of the neurons and their vast energy consumption, mitochondrial dysregulation plays an important role in the apoptotic pathway and mediates neuronal survival in several neurodegenerative diseases.^6^ Mitochondrial dysfunction has devastating effects on the BCL-2 protein family either by promoting the proapoptotic members (BAX and BAK) or inhibiting the antiapoptotic members (BCL-2 and BCL-xL), consequently leading to ATP depletion, ROS overaccumulation, mitochondrial DNA reduction, and caspase cascade activation.^34^ This is consistent with the results of previous studies, which reported that the 8AQ–uracil Cu complexes effectively decrease the percentage of apoptotic profiles and balance the proapoptotic and antiapoptotic members of the Bcl-2 family alongside the well-known antioxidant RSV.^35−37^ Moreover, in vitro studies have reported that the Cu–curcumin complex imparted neuroprotection by downregulating the NF-κB signaling pathway, upregulating BCL-2/BAX protein expression, and enhancing antioxidant enzymes with a better potency than those exhibited by the native curcumin ligand and zinc–curcumin complex.^38^

Furthermore, H_2_O_2_ is the only oxidant that functions as a second messenger in a physiologically relevant manner. Conversely, SOD2 is the first endogenous antioxidant enzyme that catalyzes the dismutation of superoxide to H_2_O_2_ in the mitochondrial matrix. Its cellular localization and function critically serve as a cytoprotective defense system against oxidative damage by scavenging the overaccumulated H_2_O_2_ through the mitochondrial ROS signaling pathway.^39^ Alongside the impaired antioxidant defense to neutralize the destructive effects of the generated ROS, cumulative oxidative stress in response to mitochondrial dysfunction causes lipid degradation and protein deposition, ultimately leading to cell death in neurodegenerative diseases. Reportedly, oxidative stress as a result of mitochondrial dysfunction occurs during the early stage of AD and is associated with elevated α-synuclein levels in Parkinson’s disease.^40^ The antioxidant properties of 8AQ-based metal complexes were supported by previous findings, which indicated that mixed ligand 8AQ–uracil metal (Cu, nickel, and manganese) complexes are active antioxidant agents.^16^

A major risk factor that drives the onset and progression of neurodegenerative disorders is accelerated aging. SIRTs are NAD^+^-dependent protein deacetylases. The modulation of SIRT activities in mammals is reportedly beneficial for various biological processes, including cellular metabolism, apoptosis, DNA repair, cell survival, immune homeostasis, and neuroprotection,^41,42^ as well as their well-known involvement in physiological stress responses. The protective effect of SIRT1/3 was hypothesized to be mediated by multiple transcriptional regulators, such as FOXO3a, tumor suppressor protein p53, peroxisome proliferator-activated receptor, and nuclear factor kappa B (NF-κB).^43^ Our findings were consistent with the above-mentioned, which revealed that the FOXO3a-mediated stress response can be specifically deacetylated by NAD^+^-dependent deacetylases (i.e., SIRT1 and SIRT3) to increase longevity,^44^ and both 8AQ–Cu–5Iu and 8AQ–Cu–5Nu can be novel alternative candidates for maintaining the healthy brain and the treatment and prevention of neurodegeneration via the SIRT1/3-FOXO3a axis. RSV is a natural stilbene extracted from grape seeds and exhibits its protective and longevity effects by activating the silent information regulator 2 (Sir2)-dependent pathway.^45,46^ Consequently, it was selected as a reference ligand for SIRT1/3 protein expression, molecular docking, and pharmacokinetic studies. In recent decades, computational tools have been used to facilitate drug development.^47^ Molecular docking has been popularly used to elucidate the possible binding modes and ligand–target interactions of candidate compounds to provide beneficial and key knowledge for successful drug design.^48^ To summarize, the molecular docking simulations in this study revealed that the two tested metal complexes occupied the SIRT1 activator binding site, allowing the formation of several binding interactions to provide a preferable binding affinity and indicating the activation potential of SIRT1. Owing to these findings, coordination with the second uracil-based ligand (5Iu or 5Nu) in the metal complex molecules can provide key functional moieties for the formation of additional chemical interactions, that is 8AQ–Cu–5Iu: halogen bonding between the I atom of the 5Iu ligand and GLU230 (Figure 5B) and 8AQ–Cu–5Nu: hydrogen bonding between the O atom of the 5Nu ligand and GLU230 (Figure 5C), which could mimic the hydrogen bonding of RSV (OH group and GLU230, Figure 5E). A unique hydrogen bonding at ARG446 via the O atom of the NO_2_ moiety of the 8AQ–Cu–5Nu was observed, while the π–alkyl interactions were noted for those formed between the benzene ring of 8AQ–Cu–5Nu, 8AQ, and RSV (Figure 5C). This could explain the lowest binding energy of 8AQ–Cu–5Nu. These findings are supported by the results of our recent study, which reported that a quinoline-based compound (nitroxoline) was a potential SIRT1 activator.^35^

Furthermore, current drug development is coping with late-stage failure owing to the poor pharmacokinetics (ADME), finally complemented by toxicity (ADMET), which constitutes important features of the candidates for successful drug development. Accordingly, *in silico* ADMET prediction has been included in the initial stages of drug discovery to increase the success rate of drug development.^49^ Results from *in silico* ADMET predictions suggested that 8AQ–Cu–5Iu, 8AQ–Cu–5Nu, and 8AQ possess drug-like properties with high gastrointestinal absorption potential, preferable bioavailability, poor skin penetration, and BBB-crossing abilities, probably due to their preferable lipophilicity and low PSA, which allow them to reach the target site within the CNS.^50,51^ Moreover, the predicted lipophilicity value of 8AQ–Cu–5Iu is higher than that of 8AQ–Cu–5Nu, probably due to the presence of the higher lipophilic iodo group of the 5Iu ligand that is absent in the NO_2_ group with an ionic charge of 5Nu. Our findings are consistent with the results of previous studies that revealed the anti-Alzheimer effect of the aminoquinoline-based metal complexes. Cu-bis(aminoquinoline) exhibits stronger Cu binding affinity than zinc binding affinity and effectively inhibits ROS production, improves BBB permeability, and recovers memory deficit in an Aβ-injected mouse model.^13^ Similarly, PA1637, a new Cu-specific bis-8-aminoquinoline, exhibited a memory-strengthening effect in nontransgenic Aβ-impaired mice by effectively reversing the episodic memory deficit with a potency comparable with that of commercial *N*-methyl-d-aspartate receptor antagonists that are clinically used to treat moderate-to-severe AD.^52^ Another metal ionophore was reported to facilitate Cu ion delivery across the BBB is a Cu-bis(thiosemicarbazone) complex whose underlying neuroprotective mechanisms included the promotion of neurite formation and elongation via the MAPK signaling pathway in PC12 cells^53^ alongside the reduction of Aβ deposition in the cognitive APP/PS1 mice.^54^ Several studies have documented that the lead compounds have failed at the late stages of clinical trials owing to the genetic variations in the metabolizing enzyme Cytochrome P450 (CYP450) family^55,56^ which plays a major role in the metabolism of a wide range of clinical drug classes, particularly CYP1A2, CYP2C9, CYP2C19, CYP2D6, and CYP3A4 isoforms.^57^ The two identifying compounds probably acted as CYP1A2 inhibitors, which suggests their potentials to produce drug–drug/food–drug interactions; hence, this consideration should be raised in the case of coadministration.

## 4 Conclusions

**Figure 6:**
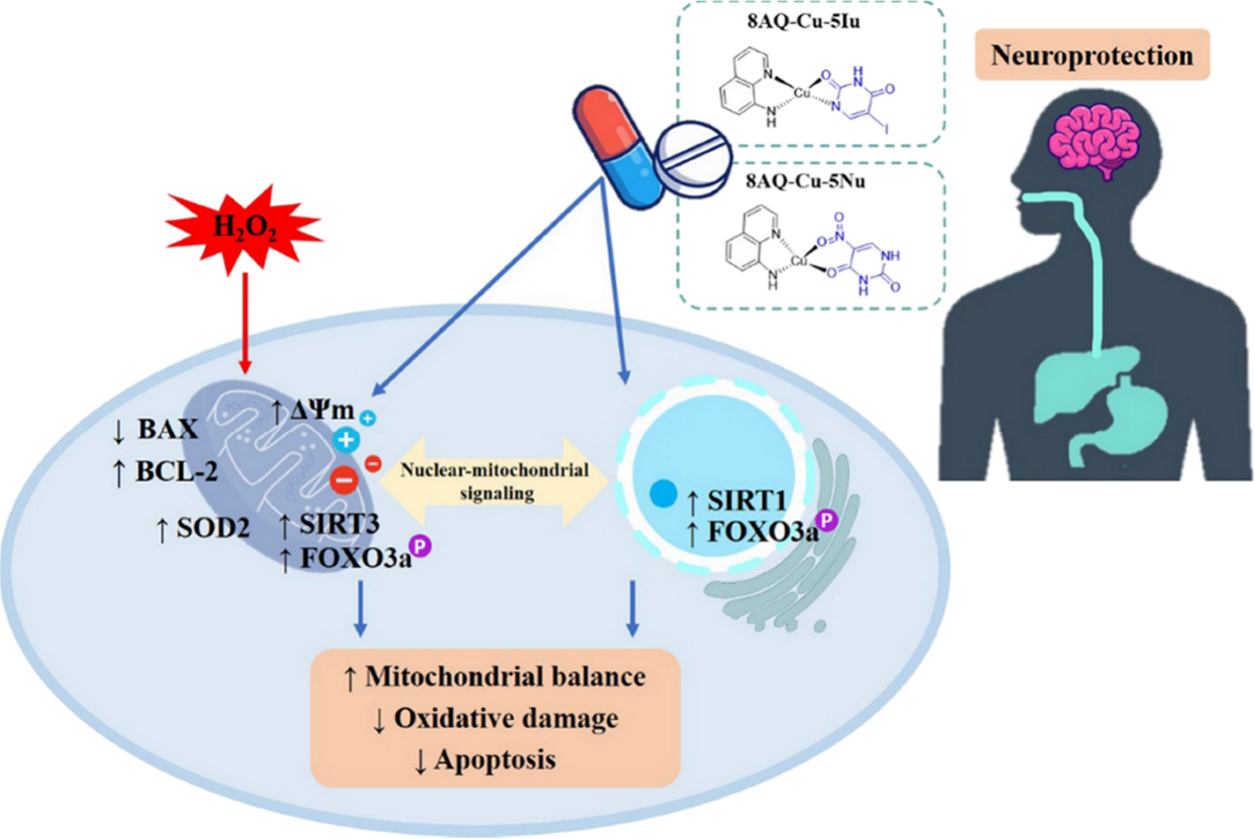
Schematic diagram illustrating the potential neuroprotective mechanism of 8AQ-based metal complexes against H_2_O_2_-induced neurodegeneration through the SIRT1/3-FOXO3a signaling pathway.

The neuroprotective effect of two 8AQ–uracil Cu complexes (8AQ–Cu–5Iu and 8AQ–Cu–5Nu) was investigated on H_2_O_2_-induced oxidative stress in human neuroblastoma SH-SY5Y cells. The results revealed that these metal complexes prevented oxidative stress-induced neuronal cell death by modulating the SIRT1/3-FOXO3a signaling pathway to impart neuroprotective effects (i.e., mitigating apoptotic cascades, activating antioxidant SOD2 enzyme, ameliorating mitochondrial dysfunction, and upregulating antiapoptotic protein expression). Furthermore, these metal complexes acted as competitive SIRT1 activators by the enhancement of the uracil-based ligand within the metal complex molecules compared with its parent ligand 8AQ. Based on *in silico* theoretical ADMET predictions, the metal complexes are drug-like compounds with preferable properties for CNS-targeting and oral administration. Overall, the investigated 8AQ-uracil metal complexes are promising compounds with a multifunctional nature (Figure 6) for further comprehensive development as a novel class of neuroprotective agents using in vivo models and clinical trials.

## 5 Methods

### 5.1 Chemicals and Antibodies

SIRT1 Assay Kit was purchased from Sigma-Aldrich (St. Louis, Missouri, USA). Annexin V & Dead Cell Assay Kit, Immobilon-PSQ polyvinylidene fluoride (PVDF) membrane, and Immobilon ECL ultra western horseradish peroxidase (HRP) substrate were obtained from Merck Millipore (Darmstadt, Germany). Moreover, MTT and 2′,7′-dichlorodihydrofluorescein diacetate (H_2_DCFDA) were purchased from Molecular Probes (Eugene, Oregon, USA). The following antibodies were used in this study: anti-SIRT1, SIRT3, FOXO3a, SOD2, BAX, BCL-2, and β-actin alongside HRP goat antimouse immunoglobulin G (IgG) and antirabbit IgG antibodies (Cell Signaling Technology, MA, USA). All reagents were of analytical reagent grade and were obtained from Sigma-Aldrich (St. Louis, Missouri, USA).

### 5.2 Synthesis of 8AQ-Based Metal Complexes

**Figure 7:**
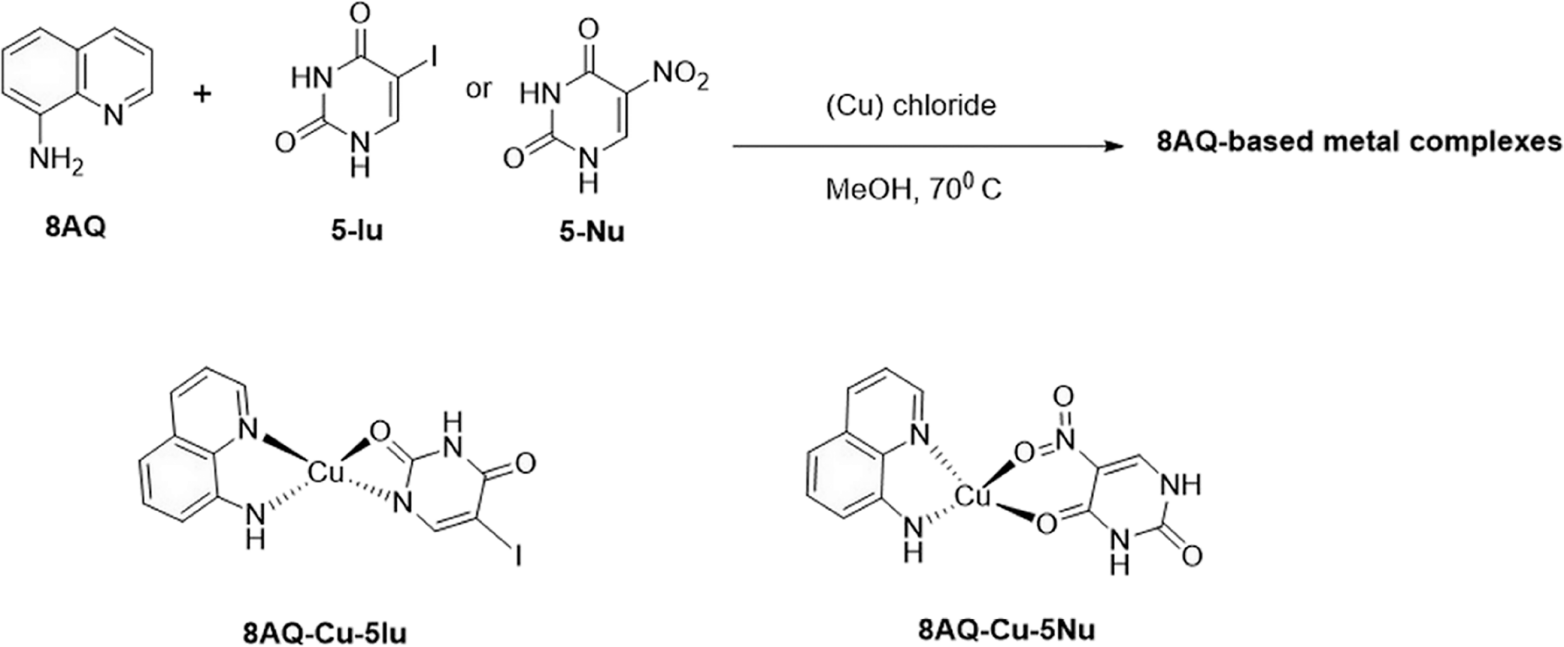
Synthesis and chemical structures of 8AQ-based metal complexes (8AQ–Cu–5Iu and 8AQ–Cu–5Nu).

According to previous studies,^15,16^ 8AQ metal complexes were synthesized from the corresponding uracil-core ligands. The mixed ligands were prepared by adding metal salts (l mmol) in methanol (2 mL) to a hot solution (70 °C) of uracil ligand (5-Iu or 5-Nu) (1 mmol) in methanol (30 mL). The mixture was heated for 45 min. A solution of 8AQ (l mmol) in methanol (2 mL) was added dropwise to the reaction mixture under heat for 1 h, following which the complexes precipitated. They were removed by filtration, washed with cold methanol, and dried *in vacuo* at room temperature. Chemical structures of the complexes were confirmed using infrared and high resolution mass spectra, magnetic moment, and melting point. The chemical structures of 8AQ–Cu complexes are presented in Figure 7.

### 5.3 Cell Culture and Treatment

SH-SY5Y human neuroblastoma cells obtained from the American Type Culture Collection (Manassas, VA, USA) were routinely maintained in Dulbecco’s modified Eagle’s medium supplemented with 1% penicillin–streptomycin and 10% inactivated fetal bovine serum (Gibco BRL, MD, USA) in a humidified atmosphere of 95% air and 5% CO_2_ at 37 °C. For all the experiments, the cells were used at ∼80% confluence. 8AQ-based metal complexes (8AQ–Cu–5Iu and 8AQ–Cu–5Nu), 8AQ, and RSV were dissolved in dimethyl sulfoxide, which had a final concentration of <0.1% (v/v).

### 5.4 Cell Viability Analysis

The cytotoxicity of 8AQ-based metal complexes was investigated by using an MTT assay. Human SH-SY5Y cells were seeded onto 96-well plates at a density of 1 × 10^5^ cells/mL and incubated overnight before detecting cell viability. Following pretreatment with various concentrations of 8AQ–Cu–5Iu, 8AQ–Cu–5Nu, 8AQ, and RSV (0.1–100 μM) for 3 h, the culture media was completely replaced with media containing 400 μM H_2_O_2_ for 24 h. Subsequently, the cells were treated with 5 mg/mL MTT in 0.1 mM phosphate-buffered saline for 3 h at 37 °C. The formazan crystals were solubilized using 0.04 N HCl–isopropanol. The optical density at 570 nm was recorded by using a microplate reader (BioTek Instruments, VT, USA).

### 5.5 Cell Apoptosis Analysis

Annexin V and 7-aminoactinomycin D were used to determine the apoptotic profiles using a flow cytometry. Briefly, SH-SY5Y cells were seeded onto 6-well plates. The cells were treated with 1 μM 8AQ–Cu–5Iu, 8AQ–Cu–5Nu, 8AQ, and RSV for 3 h and exposed to 400 μM H_2_O_2_ for 24 h. Following the end of the treatment, both floating and adherent cells were harvested and incubated with the fluorescent cocktail. The percentages of living, apoptotic, and dead cells were analyzed using a Muse cell analyzer (Merck Millipore, Darmstadt, Germany).

### 5.6 Intracellular ROS Analysis

The intracellular ROS was monitored by using a H_2_DCFDA fluorescent probe. SH-SY5Y cells were seeded onto 96-well plates and treated as described above. At the end of the treatment, the cells were treated with 10 μM carboxy-H_2_DCFDA at 37 °C for 30 min in the dark. The fluorescence of DCF at excitation and emission spectra of 492–495 and 517–527 nm was detected using a microplate reader and a fluorescence microscope (Olympus IX70, Japan).

### 5.7 MMP (ΔΨm) Analysis

The changes in MMP were measured by using rhodamine 123 (Rh-123) staining. Following the end of the treatment, the cells were incubated with 10 μM Rh-123 at 37 °C for 30 min in the dark. Subsequently, the cells were washed with phosphate-buffered saline. The fluorescence intensity was measured at excitation and emission wavelengths of 485 and 528 nm, respectively, using a microplate reader and a fluorescence microscope.

### 5.8 Western Blot Analysis

The cells were treated with 1 μM 8AQ–Cu–5Iu, 8AQ–Cu–5Nu, 8AQ, and RSV for the indicated times before harvesting the lysates for Western blotting. The protein concentration was determined by using a Bradford assay. Equivalent amounts of proteins from each group were separated using sodium dodecyl sulfate–polyacrylamide gel electrophoresis and electrotransferred onto PVDF membranes. After being blocked with 5% nonfat milk in Tris buffer containing 0.1% Tween-20 (TBST), the membranes were incubated with primary antibodies (SIRT1, SIRT3, FOXO3a, SOD2, CAT, BCL-2, BAX, and β-actin) at 4 °C overnight, followed by incubation with the corresponding HRP-conjugated secondary antibodies at room temperature for 1 h. The protein signals were visualized by using an ECL reagent. The density of each band was quantified using the ChemiDoc MP Imaging System and Image Lab Software (Bio-Rad Laboratories, Inc., CA, USA).

### 5.9 ADMET Prediction

The pharmacokinetic profiles of 8AQ-based metal complexes, 8AQ ligand, and RSV were predicted using SwissADME (http://www.swissadme.ch)^19^ and pkCSM (http://biosig.unimelb.edu.au/pkcsm/)^20^ online tools. Briefly, the two-dimensional structures of 8AQ–Cu–5Iu, 8AQ–Cu–5Nu, 8AQ, and RSV were converted to a simplified molecular-input line-entry system format to be uploaded as input files for analysis on the Web servers. A set of physicochemical parameters following Lipinski’s rule was analyzed to predict the drug-like properties of the compounds (i.e., molecules with a molecular weight of <500 Da, hydrogen bond acceptors [<10], hydrogen bond donors [<5], and lipophilicity [calculated LogP < 5]).^21,22^ Additional parameters, including PSA, rotatable bond, molar refractivity, and water solubility, were analyzed. Moreover, parameters regarding the distribution of compounds into the target site in the CNS were investigated (i.e., BBB permeation).

### 5.10 Docking Studies

Molecular docking was conducted to reveal the possible binding modalities of the 8AQ metal complexes against the target enzyme SIRT1 using AutoDock 4.2.6 software. The three-dimensional crystal structure of the SIRT1 protein was retrieved from the Protein Data Bank (PDB ID: 5BTR). The target protein was prepared by preserving an amino acid chain, and the cocrystallized ligand (RSV) was detached. The chemical structures of the investigated compounds (8AQ–Cu–5Iu, 8AQ–Cu–5Nu, and 8AQ) were drawn in two dimensions and converted to three-dimensional structures using ChemOffice 2020 (PerkinElmer Informatics, Inc.). The atomic coordinates of the metal complexes (8AQ–Cu–5Iu and 8AQ–Cu–5Nu) were optimized using Discovery Studio Visualizer 2021 (BIOVIA, Dassault Systèmes). The size of the grid box defined for AutoDock was fixed on the interface between the C- and N-terminal domains of SIRT1. The detached cocrystallized ligand (RSV) was redocked to the active site, and the RMSD value was calculated to validate the docking protocol. The docking parameters of the Lamarckian genetic algorithm^58^ were performed for 100 runs. The studied metal complexes and 8AQ ligand were docked, and the best binding pose of each compound was selected based on the binding energy compared with the RSV. Finally, the key ligand–protein interactions between the compounds and amino acid residues of the target protein were visualized using Discovery Studio Visualizer 2021.

### 5.11 Statistical Analysis

Data are expressed as the mean ± standard error. All experiments were run independently in triplicate for each treatment group. Statistical comparisons were performed using one-way analysis of variance with a Tukey–Kramer posthoc test using GraphPad Prism 6 software (GraphPad Software, CA, USA). A probability level of *P* < 0.05 was considered statistically significant.
